# Descriptive epidemiology of injuries in Japanese collegiate men’s basketball: 2013/2014 to 2019/2020

**DOI:** 10.1186/s40621-022-00368-8

**Published:** 2022-01-17

**Authors:** Yuta Sekine, Kotaro Kamada, Takeshi Koyama, Seigo Hoshikawa, Sayuri Uchino, Takayuki Komatsu

**Affiliations:** 1 Faculty of Modern Life, Teikyo Heisei University, 4-21-2 Nakano, Nakano-ku, Tokyo, 164-8530 Japan; 2 Faculty of Sports and Health Science, Daito Bunka University, 560 Iwadono, Higashimatsuyama-shi, Saitama, 355-8501 Japan; 3 Graduate School of Sport Sciences, Waseda University, 2-579-15 Mikajima, Tokorozawa-shi, Saitama, 359-1192 Japan; 4 Sports Medical Science Research Institute, Tokai University, 4-1-1 Kitakaname, Hiratsuka-shi, Kanagawa, 259-1292 Japan; 5 School of Social Informatics, Aoyama Gakuin University, 5-10-1 Fuchinobe, Sagamihara-shi, Kanagawa, 252-5258 Japan; 6 Department of Orthopaedics, Faculty of Medicine, Juntendo University, 2-1-1 Hongo, Bunkyo-ku, Tokyo, 113-8421 Japan; 7 Department of Emergency and Critical Care Medicine, Juntendo University Nerima Hospital, 3-1-10 Takanodai, Nerima-ku, Tokyo, 177-8521 Japan; 8 Department of Medicine and Science, Kanto Collegiate Basketball Federation, 27-2 Sakuragaoka-cho, Shibuya-ku, Tokyo, 150-0031 Japan

**Keywords:** Basketball, Collegiate, Injury surveillance

## Abstract

**Background:**

Basketball is one of the most played sports in the world. However, only a few studies have examined the epidemiology of Japanese collegiate men’s basketball injuries. This study investigated the incidence of injury among Japanese collegiate men’s basketball from the 2013/2014 to the 2019/2020 seasons and identified unique patterns by comparing our data with the National Collegiate Athletic Association (NCAA) men’s basketball data.

**Methods:**

Data from Japanese collegiate basketball teams of the Kanto Collegiate Basketball Federation Division I League during the 2013/2014 to 2019/2020 academic years (23 team-seasons) were used in this study. Injury rates per 1000 athlete exposures (AEs), injury proportions, and the injury rate ratio (IRR) were calculated according to the events, injury types, body parts, and common injury mechanisms. Injury rates were then compared with that from the time-loss injury data of the NCAA’s previous reports.

**Results:**

In total, 480 injuries during 97,515 AEs were reported, leading to an injury rate of 4.92 per 1000 AEs (95% CI = 4.48–5.36). The overall injury rate was higher in Japan than in the NCAA ([2009/2010–2014/2015] IRR = 1.55, 95% CI = 1.39–1.73; [2014/2015–2018/2019] IRR = 1.64, 95% CI = 1.48–1.82). Lower extremity injuries occurred most frequently (73.5%). Ankle sprain was the most common injury in Japan, with higher injury rates than in the NCAA (IRR = 2.10; 95% CI = 1.72–2.57). The injury rate of concussion was lower in Japan than in the NCAA (IRR = 0.28; 95% CI = 0.14–0.55).

**Conclusions:**

The rates of overall injury and ankle sprain were higher and that of concussion was lower in Japan than in the NCAA. These results suggested the existence of international differences in the pattern or features of injuries in basketball players.

## Background

As a high-intensity sport, basketball is characterized by high aerobic and anaerobic demands, continuous changes in direction, accelerations and decelerations, jumps, sprints, contacts, and specific skills (Ben Abdelkrim et al. 2007; McInnes et al. 1995). The nature of basketball, such as changes in direction, player contact, repetitive jumping, and landing activities, might affect the incidence of lower extremity injury (Zuckerman et al. 2018), particularly ankle sprain (Tummala et al. 2018).

Various epidemiological studies on sports-related injuries have been reported from the injury surveillance program of the National Collegiate Athletic Association (NCAA-ISP) (Zuckerman et al. 2018; Tummala et al. 2018; Clifton et al. 2018; Dick et al. 2007a; Morris et al. 2021). The studies from the NCAA-ISP emphasize a high level of evidence-based practices related to injury prevention and are a vital resource for further research (Curtis et al. 2008; Silvers-Granelli et al. 2015). In Japan, the Japan Association for University Athletics and Sport (UNIVAS) was established in 2019 by the Japan Sports Agency, an external bureau of the Ministry of Education, Culture, Sports, Science and Technology (MEXT) (Japan Association for University Athletics and Sport 2021). One of the chief projects of the UNIVAS is to improve the environment for collegiate athletic activities and to increase engagement in sports, safely and securely. To achieve these objectives, surveys and research on the aspects related to sports activity-related accidents are required.

A total of 597,375 basketball players registered with the Japan Basketball Association in 2019 comprised over 8000 collegiate men (Japan Basketball Association 2021). To prevent injury and illness and to improve the athletic performance of the Japanese collegiate basketball players, the Department of Medicine and Science attached to the Kanto Collegiate Basketball Federation (KCBF) was established, comprising the area including the Tokyo, Kanagawa, Chiba, Saitama, Gunma, Ibaraki, and Tochigi prefectures (Kanto Collegiate Basketball Federation 2021). Currently, there have been no epidemiological studies on Japanese collegiate basketball players. Although one epidemiological study including elementary school mini-basketball players with a mean age of 10.9 ± 1.0 was reported, the rules and standards for mini-basketball vastly differ from those of general basketball, including ball size, goal height, and game time (Kuzuhara et al. 2016). Moreover, the characteristics of injuries in the childhood category alone was unidentifiable. For preventive intervention research in basketball players, accurate epidemiological data are needed. In addition, an international comparison with the results of previous studies might help to find and recognize the current medical issues surrounding Japanese basketball players. Therefore, we aimed to describe the incidence of injuries in Japanese collegiate men’s basketball from the 2013/2014 to the 2019/2020 seasons. We further aimed to investigate unique patterns by comparing our data with the NCAA’s men’s basketball data, reported in previous researches (Zuckerman et al. 2018; Morris et al. 2021).

## Methods

### Data source

Data managed by the Department of Medicine and Science of the KCBF were used in this study. The duration of the investigation was from the 2013/2014 to the 2019/2020 academic years in Japan (April 1st–March 31st). A total of seven teams from the KCBF Division I League, consisting of 10 (until 2017) to 12 teams (2017-present), participated in the investigation. Since some teams were unable to continue the survey due to factors such as dropping out of the survey (2 teams) and replacing divisions (1 team), this study was conducted using mixed data (23 team-seasons). This study was approved by the Human Ethics Review Committee of Teikyo Heisei University (No. R01-080-1). The study was conducted according to the tenets of the Declaration of Helsinki.

### Data collection

The injury and exposure data collected under the supervision of an athletic trainer certified by the Japan Sports Association in each team (all of them were employed part-time) were aggregated for each season. Data were recorded in a pre-designed and unified electronic spreadsheet, which were collected at the end of each season. Injuries that occurred during basketball games or basketball-specific practices (e.g., shooting drills, offensive or defensive moves, and scrimmages) were included in the study. Any injuries that occurred in weight training or conditioning sessions (e.g., sprint training, agility training, and plyometrics) and illnesses were excluded. Thus, we excluded a total of 4 injuries during weight training and conditioning and 51 illnesses.

### Definitions

Based on previous studies Kuzuhara et al. (2016), Dick et al. (2007b), an injury was defined as any event that (1) occurred as a result of participation in regular practice or competition in sports, (2) caused the player to seek medical care from a physician or alternative medical specialist, or (3) resulted in the restriction of student-athlete participation or performance for one or more calendar days since the day of injury. Time loss was one of the criteria used to describe the severity of health problems in sports in the present study (Bahr et al. 2020). To compare our data with the severe injuries reported in the previous study (Zuckerman et al. 2018), injuries that required > 3 weeks to heal and allow the player to regain complete fitness for playing basketball or injuries that led to player retirement were defined as severe injuries. Athlete exposure (AE) was defined as one athlete participating in the practice or official competition organized by KCBF and the All Japan University Basketball Federation, wherein the player was exposed to the possibility of athletic injury, regardless of the time of participation. The player who warmed up before the match but did not play was not considered an AE.

Body parts, injury types, and mechanisms were classified as follow (Table 1). To compare with previous research, isolated or a combination of anterior cruciate ligament (ACL), posterior cruciate ligament, collateral ligament (medial or lateral, not differentiated), or meniscus (medial or lateral, not differentiated) injury was also categorized as “knee internal derangement.” (Zuckerman et al. 2018).

**Table 1:** Classification of body parts, injury types, and mechanisms

Body parts	Injury types	Mechanisms of injury
Head/face	Sprain	Contact (with another player)
Neck	Strain	Contact (with an object)^c^
Shoulder	Contusion	No contact
Arm/elbow	Concussion	Overuse
Hand/wrist	Fracture	
Trunk^a^	Dislocation/subluxation	
Hip/groin	Laceration	
Upper leg	Tendonitis	
Knee	Nerve injury	
Lower leg	Cartilage injury^b^	
Ankle	Other	
Foot		

^a^Including the chest, abdomen, upper back, and lower back ^b^Including meniscus injury ^c^Including the ball, surface, equipment, etc.

### Statistical analyses

The injury rate was calculated as the number of injuries per 1000 AEs. In the injury rate ratio, all 95% CIs, not including 1.0, were considered statistically significant. The calculation of injury rates and rate ratios was analyzed with 95% confidence intervals (CIs) using Microsoft® Excel for Mac (version 16.45, Microsoft Corp, Redmond, WA) (Knowles et al. 2006). The distribution of the mechanisms of injury and proportion of severity in each mechanism of injury were compared using the *χ*^2^ test, using SPSS® software (version 27.0; IBM Corporation, Armonk, NY, USA). The alpha level was set to *p* < 0.05. Following analysis, we attempted to compare our data with the reported injury data on the NCAA men’s basketball injuries from 2009/2010–2014/2015 and 2014/2015–2018/2019 (Zuckerman et al. 2018; Morris et al. 2021). Common injuries and severe injuries were also compared with those published in the previous study (Zuckerman et al. 2018). Since non-time-loss injuries, which were defined as injuries resulting in participation restriction for < 24 h, were not recorded in the present study, only time-loss injuries reported in previous studies were included for comparison. Injury rates and 95% CIs were recalculated and applied from reported AEs and number of time-loss injuries.

## Results

### Overall injury rates

Over the period of 7 years, a total of 480 injuries across 23 team-seasons were reported, of which 346 (72.1%) occurred in practice, 130 (27.1%) occurred in competition, and 4 (0.8%) had missing event information (Table 2). These injuries occurred during 97,515 AEs (practice: 89,559 AEs; competition: 7956 AEs), and a total of 87 (18.1% of overall injuries) were considered severe injuries (> 21 days lost), one of which led to forced medical retirement. A total of 57 (65.5% of severe injuries) occurred in practice, 29 (33.3% of severe injuries) occurred in competition, and 1 (0.2%) was missing the event information. Injury rates in competition were higher than those in practice among all injuries (IRR = 4.23, 95% CI = 3.46–5.17) and severe injuries (IRR = 5.73, 95% CI = 3.66–8.96).

**Table 2:** Injury rates and 95% CIs by the events in Japanese collegiate men’s basketball, 2013/2014–2019/2020 and comparison with NCAA men's basketball

	Japan		NCAA				Japan versus NCAA	
			2009/2010–2014/2015		2014/2015–2018/2019		2009/2010–2014/2015	2014/2015–2018/2019
	*n*	IR and 95% CI (per 1000 AEs)	*n*	IR and 95% CI (per 1000 AEs)	*n*	IR and 95% CI (per 1000 AEs)	IRR (95% CI)	IRR (95% CI)
*Practice*								
Injuries	346	3.86 (3.46–4.27)	635	2.80 (2.58–3.02)	950	2.59 (2.43–2.76)	1.38 (1.21–1.57)*	1.49 (1.32–1.68)*
Severe injuries	57	0.64 (0.47–0.80)	65	0.29 (0.22–0.36)			2.19 (1.60–3.01)*	
*Competition*								
Injuries	130	16.34 (13.53–19.15)^†^	286	4.56 (4.03–5.09)	482	4.31 (3.93–4.69)	3.58 (2.91–4.41)*	3.79 (3.12–4.60)*
Severe injuries	29	3.65 (2.32–4.97)^†^	52	0.83 (0.60–1.05)			4.39 (3.00–6.43)*	
*Overall*^a^								
Injuries	480	4.92 (4.48–5.36)	921	3.18 (2.98–3.39)	1432	3.00 (2.84–3.15)	1.55 (1.39–1.73)*	1.64 (1.48–1.82)*
Severe injuries	87	0.89 (0.71–1.08)	117	0.40 (0.33–0.48)			2.23 (1.69–2.94)*	

AEs; athlete exposure(s): *Practice* = 89,559, *Competition* = 7,956, CI; confidence interval, IR; injury rate, IRR; injury rate ratio

^a^*Overall* injuries do not equal sum of *Practice* and *Competition* injuries due to four injuries missing the event information

^*^Japan versus the NCAA data (Zuckerman et al. 2018; Morris et al. 2021) injury rate ratio > 1.00 and does not include 1.00 in the 95% CI

^†^Competition versus Practice injury rate ratio > 1.00 and does not include 1.00 in the 95% CI

### Mechanisms of injury

**Fig. 1:**
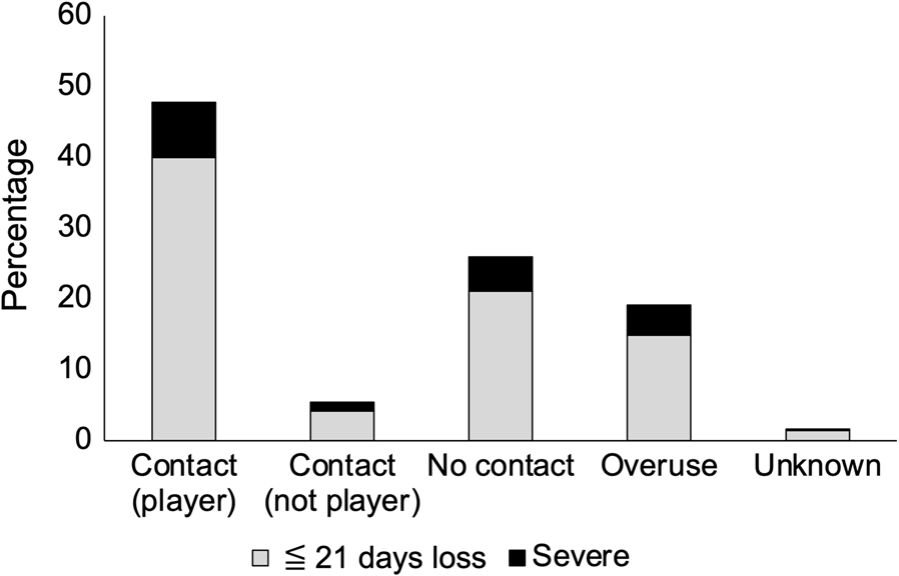
The distribution of the mechanisms of injury. Note: One injury in each of contact (player), contact (not player), no contact, and overuse had no time-loss recorded

Figure 1 shows the distribution of the mechanisms of injury for all injuries and the proportion of severe injuries in each mechanism of injury. The most common mechanism of injury was contact with another player (*n* = 228, 47.5%), followed by no contact (*n* = 124, 25.8%), overuse (*n* = 93, 19.4%), and contact with an object (*n* = 27, 5.6%) (*χ*^2^ = 320.02, *p* < 0.001). A total of 53.1% of injuries were contact-related (*n* = 255). The proportion of severe injuries was as follows: overuse (*n* = 21, 22.6%), contact with an object (*n* = 6, 22.2%), no contact (*n* = 23, 18.5%), and contact with another player (*n* = 37, 16.2%).

### Injuries by body part

Lower extremity (including hip/groin, upper leg, knee, lower leg, ankle, and foot) injuries accounted for the majority of total injuries (73.5%) (Table 3). In particular, ankle (35.8%), upper leg (12.1%), and trunk (11.0%) injuries were the most commonly reported. Injury rates in all body parts, except for the arm/elbow and hip/groin, were higher in competitions than during practice. Knees had the most severe injuries (40.8% of all knee injuries; median, range of days lost = 77, 24–500).

**Table 3:** Injury counts, rates (per 1000 Athletes Exposures), and percentage of severity by body part and type of event in Japanese collegiate men’s basketball, 2013/2014–2019/2020

	Practice		Competition		Overall ^a^		
	*n* (%)	IR and 95% CI (per 1000 AEs)	*n* (%)	IR and 95% CI (per 1000 AEs)	*n* (%)	IR and 95% CI (per 1000 AEs)	% Severe (median of days lost, range)
Head/face	13 (3.8)	0.15 (0.07–0.22)	12 (9.2)	1.51 (0.65–2.36)*	25 (5.2)	0.26 (0.16–0.36)	8 (29.5, 28–31)
Neck	2 (0.6)	0.02 (0.0–0.05)	0	0	2 (0.4)	0.02 (0.0–0.05)	50 ^b^
Shoulder	11 (3.2)	0.12 (0.05–0.20)	9 (6.9)	1.13 (0.39–1.87)*	20 (4.2)	0.21 (0.12–0.29)	30 (93.5, 42–180)
Arm/elbow	7 (2.0)	0.08 (0.02–0.14)	2 (1.5)	0.25 (0.0–0.60)	9 (1.9)	0.09 (0.03–0.15)	33.3 (25, 25–60)
Hand/wrist	10 (2.9)	0.11 (0.04–0.18)	8 (6.2)	1.01 (0.31–1.70)*	18 (3.8)	0.18 (0.10–0.27)	33.3 (49, 37–81)
Trunk ^c^	40 (11.5)	0.45 (0.31–0.59)	11 (8.4)	1.38 (0.57–2.20)*	53 (11.0)	0.54 (0.40–0.69)	15.1 (25, 24–44)
Hip/groin	13 (3.8)	0.15 (0.07–0.22)	1 (0.8)	0.13 (0.0–0.37)	14 (2.9)	0.14 (0.07–0.22)	0
Upper leg	44 (12.7)	0.49 (0.35–0.64)	14 (10.8)	1.76 (0.84–2.68)*	58 (12.1)	0.59 (0.44–0.75)	15.5 (36,22–120)
Knee	34 (9.8)	0.38 (0.25–0.51)	15 (11.5)	1.89 (0.93–2.84)*	49 (10.2)	0.50 (0.36–0.64)	40.8 (77, 24–500)
Lower leg	26 (7.5)	0.29 (0.18–0.40)	8 (6.2)	1.01 (0.31–1.70)*	34 (7.1)	0.35 (0.23–0.47)	17.6 (36.5, 30–65)
Ankle	127 (36.7)	1.42 (1.17–1.66)	43 (33.1)	5.40 (3.79–7.02)*	172 (35.8)	1.76 (1.50–2.03)	13.4 (30, 22–104)
Foot	19 (5.5)	0.21 (0.12–0.31)	7 (5.4)	0.88 (0.23–1.53)*	26 (5.4)	0.27 (0.16–0.37)	15.4 (95, 30–139)

AEs; athlete exposure(s): *Practice* = 89,559, *Competition* = 7,956, CI; confidence interval, IR; injury rate, IRR; injury rate ratio

*Competition versus Practice injury rate ratio > 1.00 and does not include 1.00 in the 95% CI

^a^Overall injuries do not equal the sum of Practice and Competition injuries due to four injuries missing the event information (two are in the ankle and two are in the trunk)

^b^One player retired due to the injury

^c^Including the chest, abdomen, upper back, and lower back

### Injury types

All injury rates except for those of tendonitis were higher in competitions than that during practice (Table 4). Sprains (44.8%), contusions (13.5%), and strains (10.0%) accounted for the largest proportion of overall injuries. Cartilage injury was noted to be the most severe injury (72.7% of all cartilage injuries; median, range of days lost = 60.5, 45–109).

**Table 4:** Injury counts, percentages and rates (per 1000 Athletes Exposures) by type of injury and event in Japanese collegiate men’s basketball, 2013/2014–2019/2020

	Practice		Competition		Overall		
	*n* (%)	IR and 95% CI (per 1000 AEs)	*n* (%)	IR and 95% CI (per 1000 AEs)	*n* (%)	IR and 95% CI (per 1000 AEs)	% Severe (median of days lost, range)
Sprain	155 (44.8)	1.73 (1.46–2.00)	60 (46.2)	7.54 (5.63–9.45)*	215 (44.8)	2.20 (1.91–2.50)	15.8 (30, 22–500)
Strain	39 (11.3)	0.44 (0.30–0.57)	9 (6.9)	1.13 (0.39–1.87)*	48 (10.0)	0.49 (0.35–0.63)	16.7 (36, 22–82)
Contusion	38 (11.0)	0.42 (0.29–0.56)	27 (20.8)	3.39 (2.11–4.67)*	65 (13.5)	0.67 (0.50–0.83)	9.2 (34, 24–120)
Concussion	5 (1.4)	0.06 (0.01–0.10)	4 (3.1)	0.50 (0.01–1.00)*	9 (1.9)	0.09 (0.03–0.15)	11.1 (22)
Fracture	19 (5.5)	0.21 (0.12–0.31)	8 (6.1)	1.01 (0.31–1.70)*	27 (5.6)	0.28 (0.17–0.38)	51.9 (49, 25–139)
Dislocation/subluxation	7 (2.0)	0.08 (0.02–0.14)	6 (4.6)	0.75 (0.15–1.36)*	13 (2.7)	0.13 (0.06–0.21)	30.8 (96, 87–180)
Laceration	6 (1.7)	0.07 (0.01–0.12)	6 (4.6)	0.75 (0.15–1.36)*	12 (2.5)	0.12 (0.05–0.19)	0
Tendonitis	31 (9.0)	0.35 (0.22–0.47)	4 (3.1)	0.50 (0.01–1.00)	35 (7.3)	0.36 (0.24–0.48)	17.1 (32, 25–83)
Nerve injury	8 (2.3)	0.09 (0.03–0.15)	0	0	8 (1.7)	0.08 (0.03–0.14)	37.5 (24, 24–25) ^b^
Cartilage injury	7 (2.0)	0.08 (0.02–0.14)	3 (2.3)	0.38 (0.0–0.80)*	11 (2.3)^a^	0.11 (0.05–0.18)	72.7 (60.5, 45–109)
Other	31 (9.0)	0.35 (0.22–0.47)	3 (2.3)	0.38 (0.0–0.80)	37 (7.7)^a^	0.38 (0.26–0.50)	10.8 (65.5, 33–96)

AEs; athlete exposure(s): *Practice* = 89,559, *Competition* = 7956, CI; confidence interval, IR; injury rate, IRR; injury rate ratio

*Competition versus Practice injury rate ratio > 1.00 and does not include 1.00 in the 95% CI

^a^Overall injuries do not equal sum of Practice and Competition injuries due to missing event information

^b^One player retired due to the injury

### Common injuries

Ankle sprains were the most common in the present study (Table 5). Other common injuries included the following in order of increasing value: lower back injuries, thigh contusions, knee internal derangements, and hamstring strains. The rates of ankle sprains, thigh contusions, and knee internal derangements were higher in competitions than during practice.

**Table 5:** Common injuries in Japanese collegiate men’s basketball, 2013/2014–2019/2020

Injury	Practice		Competition		Overall		
	*n* (%)	IR and 95% CI (per 1000 AEs)	*n* (%)	IR and 95% CI (per 1000 AEs)	*n* (%)	IR and 95% CI (per 1000 AEs)	% Severe (median of days lost, range)
Ankle sprain	120 (34.7)	1.34 (1.10–1.58)	43 (33.1)	5.40 (3.79–7.02)*	163 (34.0)	1.67 (1.41–1.93)	11.7 (30, 22–104)
Lower back injury^a^	36 (10.4)	0.40 (0.27–0.53)	7 (5.4)	0.88 (0.23–1.53)	45 (9.3)	0.46 (0.33–0.60)	18.2 (25, 24–44)
Thigh contusion	25 (7.2)	0.28 (0.17–0.39)	12 (9.2)	1.51 (0.65–2.36)*	37 (7.7)	0.38 (0.26–0.50)	8.1 (36, 32–120)
Knee internal derangement	14 (4.0)	0.16 (0.07–0.24)	9 (7.0)	1.13 (0.39–1.87)*	23 (4.8)	0.24 (0.14–0.33)	65.2 (106, 30–500)
Hamstring strain	13 (3.8)	0.15 (0.07–0.22)	2 (0.5)	0.25 (0.0–0.60)	15 (3.1)	0.15 (0.08–0.23)	33.3 (36, 22–82)

AEs; athlete exposure(s): *Practice* = 89,559, *Competition* = 7956, CI; confidence interval, IR; injury rate, IRR; injury rate ratio

*Competition versus Practice injury rate ratio > 1.00 and does not include 1.00 in the 95% CI

^a^Overall injuries do not equal sum of Practice and Competition injuries due to 2 lower back injuries missing the event information

### Comparison with the NCAA data (overall injury rates, common injuries)

The overall injury rates in Japan were higher than those reported by the NCAA for the periods of 2009/2010–2014/2015 and 2014/2015–2018/2019 ([2009/2010–2014/2015]: practice IRR = 1.38, 95% CI = 1.21–1.57; competition IRR = 3.58, 95% CI = 2.91–4.41; overall IRR = 1.55, 95% CI = 1.39–1.73) and [2014/2015–2018/2019]: practice IRR = 1.49, 95% CI = 1.32–1.68; competition IRR = 3.79, 95% CI = 3.12–4.60; overall IRR = 1.64, 95% CI = 1.48–1.82). Severe injury rates were also higher in Japan than in the NCAA (practice IRR = 2.19, 95% CI = 1.60–3.01, competition IRR = 4.39, 95% CI = 3.00–6.43, and overall IRR = 2.23, 95% CI = 1.69–2.94) (Table 2). Ankle sprains constituted the highest proportion of injuries in Japan, as in the NCAA; however, the rate was higher in Japan than that reported by the NCAA (IRR = 2.10, 95% CI = 1.72–2.57) (Table 6). The concussion rate in Japan was less than that reported by the NCAA (IRR = 0.28, 95% CI = 0.14–0.55).

**Table 6:** Comparison of common injuries in Japanese collegiate and NCAA men’s basketball

Injury	Japan		NCAA		Japan versus NCAA
	*n*	IR and 95% CI (per 1000 AEs)	*n*	IR and 95% CI (per 1000 AEs)	IRR and 95% CI
Ankle sprain	163	1.67 (1.41–1.93)	230	0.80 (0.69–0.90)	2.10 (1.72–2.57) *
Hand/wrist sprain	7	0.07 (0.02–0.13)	30	0.10 (0.07–0.14)	0.69 (0.30–1.58)
Concussion	9	0.09 (0.03–0.15)	97	0.34 (0.27–0.40)	0.28 (0.14–0.55)^†^
Hip/groin strain	5	0.05 (0.01–0.10)	30	0.10 (0.07–0.14)	0.49 (0.19–1.27)
Knee internal derangement	23	0.24 (0.14–0.33)	59	0.20 (0.15–0.26)	1.16 (0.71–1.87)

AEs; athlete exposure(s): Overall in Japan = 97,515, CI; confidence interval, IR; injury rate, IRR; injury rate ratio

* Japan versus the NCAA data reported by Zuckerman et al. (2018) injury rate ratio > 1.00 and does not include 1.00 in the 95% CI

^†^The NCAA data reported by Zuckerman et al. (2018) versus Japan injury rate ratio > 1.00 and does not include 1.00 in the 95% CI

## Discussion

This study primarily aimed to describe the incidence of injuries in Japanese collegiate men’s basketball between the 2013/2014 and 2019/2020 seasons. Injury rates were four times as high in competitions as in practices. This result corresponded to previous reports that concluded that intensity demands are greater during competitions than during practice (Clifton et al. 2018; Hootman et al. 2007). In particular, the severe IRR might accentuate the high activity intensity in competitions rather than in practice (IRR = 5.73, 95% CI = 3.66–8.96). Practice was not classified in our investigation, although it included a variety of intensity contents (e.g., shooting drill, offensive or defensive moves, and scrimmages). The subdivision of events in practice would allow us to clarify the proportion of injuries.

Compared with previous reports (Zuckerman et al. 2018; Morris et al. 2021), the overall injury rates were 1.55 to 1.64 times as high in Japan as those reported in the NCAA. Additionally, overall severe injury (time lost for more than 21 days) in Japan was 2.23 times higher than that reported in the NCAA (Zuckerman et al. 2018). A previous study concluded that more skilled athletes might be at a greater risk of potential injury (Clifton et al. 2018). Furthermore, the height and strength of basketball players would be directly proportionate to the impact of the force generated during play, which would thereby potentially increase the risk of injury (Clifton et al. 2018). The present results were inconsistent with those of previous studies, and the reasons were unclear. Some limitations in the interpretation of timeloss as severity were stated, since this consisted of several factors (unique individual pattern, recovery process, etc.) (Chandran et al. 2020). The fact that Japanese collegiate basketball players have little opportunity to undergo an appropriate recovery process during the time-loss period might have influenced the results of the present study. Insufficient medical support systems for recovery processes in Japanese collegiate athletes might also be one of the factors of the present results. Clifton et al. discussed that the possibility of lesser implementation of injury prevention strategies, such as coverage by full-time athletic trainers, in schools with relatively fewer resources might have resulted in their higher injury rate (Clifton et al. 2018). Our results indicate the necessity to recognize the deficiency of the availability of medical support for collegiate athletes in Japan. To clarify these inferences, further investigation should be conducted to confirm the details of the medical support system in Japanese collegiate athletes and preventive efforts for sports injuries, and to evaluate the role of potential determinants that led to time loss (Chandran et al. 2020).

### Injury mechanisms

The leading cause of injury was contact with another player (*n* = 228, 47.5%), and no contact was the second most common injury mechanism (*n* = 124, 25.8%). The present results support previous findings in collegiate men’s basketball players (Clifton et al. 2018; Dick et al. 2007a). Details of the mechanism of injury may provide an important basis for injury prevention strategies (Silvers-Granelli et al. 2015; Longo et al. 2012; Omi et al. 2018). On the other hand, the classification of indirect contact mechanism (defined as any injury sustained through external forces that did not directly cause the injury but influenced the natural process of movement) (Luig et al. 2020) should also be considered due to the characteristics of basketball injuries. In particular, injuries caused by indirect external forces might be characteristic of basketball due to its specific activities (e.g., landing from aerial contact during a rebound or shot). The definition of the injury mechanism needs to be further clarified in future studies.

### Body site

Lower extremity injuries occurred most frequently (*n* = 353, 73.5% of overall injuries). Similar to previous studies (Zuckerman et al. 2018; Clifton et al. 2018; Morris et al. 2021), the ankle was the most frequently injured part in this study on Japanese basketball players (35.8% of overall injuries). However, injury of the knee, which was the second most frequent injury in NCAA men’s basketball players (Zuckerman et al. 2018; Clifton et al. 2018; Morris et al. 2021), was not the second most common injury after injury of the ankle in Japan. A previous study examined the predictors of the knee valgus angle, which is a risk factor for ACL injury during drop-jump landing and reported the possibility that body height was associated with the knee valgus angle during landing (Nilstad et al. 2015). The authors concluded that a greater body height, which correlated with femur and tibia length, provided longer lever arms and greater demands of strength to control the knee joint. This was inferred as one of several potential factors influencing the lesser proportion of knee injuries in Japanese collegiate men’s basketball players (189.0 ± 7.0 cm) (Koyama et al. 2020) on account of body height, which was less than that of the NCAA men’s basketball players (197.6 ± 7.1 cm) (Heishman et al. 2020).

In the injury surveillance between the 2013/2014 and 2019/2020 seasons, only two cases of neck injuries that occurred in practice were reported. In one case, the player reportedly was forced to retire due to injury. Our results suggest the necessity to be mindful not only of the magnitude of the injury rate, but also of the possibility of serious incidence, even if it is lower than others. This awareness is also required while preparing for emergencies for all the staff, as well as spectators, on the basketball court.

### Injury type

Previous studies have reported that the most frequent injury types were sprain, strain, and concussion in NCAA men’s basketball (Clifton et al. 2018; Dick et al. 2007a). In Japan, sprain and strain were most frequent, as in the NCAA, although the frequency of concussion was different. The rate of concussion in Japan was significantly lower than that in the NCAA (Table 5). We believe that the concussion rate is not simply affected by the difference in activity intensity. In the NCAA, increasing sports-related concussions were observed after the new concussion policy (Baugh et al. 2015) was adopted, and it was concluded that the increased sensitivity to concussion in players and medical personnel and reporting might reflect an increase in concussion incidence (Zuckerman et al. 2015). Moreover, there is a possibility that in this study in Japan, such injuries should have been considered as a “concussion” as they may have been overlooked due to variability in clinical presentation. The rate of concussion in our study highlights the importance of judging appropriately. In 2015, the Japanese Society of Clinical Sports Medicine released the suggestion of first-aid for head injuries, which included contents based on recent findings in head injuries to help the decisions and actions of people who stand by the athletes (i.e., coaches and parents) (Japanese Society of Clinical Sports Medicine 2021). To continue the investigation, we could obtain more accurate findings for further research, addressing the incidence, prevention, and evaluation of public awareness of the importance of head injury.

### Common injury

Consistent with previous epidemiologic studies from the NCAA of collegiate basketball players, ankle sprain was the most frequent injury in Japan, as well. Interestingly, however, the incidence rate was 2.10 times as high in Japan as that reported in the NCAA. This apparent difference indicates the necessity to improve the recognition, prevention, and appropriate treatment of ankle sprains in Japanese basketball players. This is especially true for those below the collegiate category, which urgently needs improvement. In addition to prophylactic injury prevention strategies such as neuromuscular proprioceptive training and external ankle support (ankle bracing and taping) (Tummala et al. 2018; McGuine and Keene 2006; Taylor et al. 2015; Riva et al. 2016), screening the history of ankle injury is the best way to identify risk factors (Tummala et al. 2018) since a history of ankle sprains is the most common risk factor for recurrence (with an almost fivefold increased risk) (McKay et al. 2001). The high incidence of ankle sprains in Japan might be due to the environment surrounding young athletes. Moreover, some players, who had an ankle sprain, might have developed chronic ankle instability, which is characterized by recurrent ankle sprain (Hertel and Corbett 2019). In Japan, the school administration, staff, and faculty are unequipped with knowledge and skills regarding the prevention and management of sport-specific emergencies. This is because the current school safety guidelines set forth by the MEXT focus on community safety, traffic safety, and natural disaster safety, without considerations for sport-specific safety. Due to concerns of catastrophic sports incidents associated with the current situation, national and organizational actions to reconsider the current structure of school-organized sports and to improve access to medical personnel during school-organized sports are required (Hosokawa et al. 2021). Our results showed that the higher incidence of ankle sprain in Japan might be influenced by the history or process of treatment in younger generations. This study did not examine the history of ankle sprains, which is a potential risk factor. For an appropriate prevention program for the extremely high incidence of ankle sprain in Japanese collegiate basketball players, an investigation of the history of ankle sprain and prospective studies on the injury in youth basketball players are required.

## Limitations

To the best of our knowledge, this study is the first descriptive epidemiological study of Japanese collegiate men’s basketball players. However, the surveillance size (7 years, 23 team-seasons, 97,515 AEs) of the present study was less than that in the NCAA reports during 2009/2010 to 2014/2015 (6 years, 176 team-seasons, 289,406 AEs) (Zuckerman et al. 2018) and 2014/2015 to 2018/2019 (5 years, 276 participating programs, 478,150 AEs) (Morris et al. 2021). Due to the diversity in sports and the activities that comprise them, there is no single approach to appropriately express risk for all sports injury surveillance projects (Bahr et al. 2020). The adjustment for injury rate ratios to explain the differences in the population and the characteristics between collegiate basketball players in Japan and the NCAA would be necessary for further investigation. Furthermore, because time-based measures better facilitate comparisons across sports and can affect the calculation and interpretation of estimated injury rates, it is important to consider challenges associated with the detailed measurement of time spent at risk for injury (Morris et al. 2021; Bahr et al. 2020). According to the International Olympic Committee consensus statement, the duration of the period for which an athlete is unable to train/play is called time loss, which is included in assessing the severity of the health problem (Bahr et al. 2020). Some limitations when considering time loss as a measure of severity were suggested (no standards of severity, the unique individual of the timing of return to play, etc.), persuading and advocating the need to directly examine time loss (Chandran et al. 2020). For these reasons, consideration must be given when defining severity in further surveillance, other than time loss alone. Since the epidemiological data would be an important outcome for further intervention investigation, expanding the scale of the survey and creating an appropriate environment for conducting it are necessary. We did not record the details of the activity, mechanism, or events during the injury. In particular, ankle injury, which had the highest incidence in this study, might be attributed to the nature of basketball, which involves rapid changes in direction, contacts, repetitive jumping, and landing activities (Tummala et al. 2018). Moreover, since non-time-loss injuries (participation restriction for < 24 h) were not included in the present study, data on these injuries were not collected. The inclusion of non-time-loss injuries to account for the full breadth of injuries sustained by basketball athletes has also been noted (Zuckerman et al. 2018). The definition of each item should be carefully reviewed to develop additional research.

## Conclusion

We aimed to describe the incidence of injuries in Japanese collegiate men’s basketball from the 2013/2014 to the 2019/2020 season and to investigate the unique patterns emerging from comparing the data with the men’s NCAA basketball data, from their current research. The findings that the injury rate during competition is higher than that during practice, and that ankle sprain was the most common injury, were consistent. However, the rate of overall injury and the rate of ankle sprains were 1.55 to 1.64 times and 2.10 times as high in Japanese collegiate men’s basketball players as those in NCAA men’s basketball players, respectively. Moreover, the rate of concussion was 0.28 times as less in Japan as that reported in the NCAA. We concluded that these results may have been influenced by the environment surrounding basketball players and the level of medical support available for various generations of Japanese athletes. Our results provide a foundation for future research aimed at injury prevention and suggest the urgent necessity to improve the medical support systems to protect basketball players from injury.

## Data Availability

The datasets used and analyzed during the current study are available from the corresponding author on reasonable request.

## Abbreviations

ACL: Anterior cruciate ligament
AE: Athlete exposure
IRR: Injury rate ratio
KCBF: Kanto Collegiate Basketball Federation
MEXT: Ministry of Education, Culture, Sports, Science and Technology
NCAA: National Collegiate Athletic Association
NCAA-ISP: National Collegiate Athletic Association Injury Surveillance Program
UNIVAS: Japan Association for University Athletics and Sport
95% CI: 95% Confidence interval
